# Nuclear receptor co-activators and HER-2/neu are upregulated in breast cancer patients during neo-adjuvant treatment with aromatase inhibitors

**DOI:** 10.1038/sj.bjc.6605324

**Published:** 2009-09-15

**Authors:** M Hauglid Flågeng, L L Haugan Moi, J M Dixon, J Geisler, E A Lien, W R Miller, P E Lønning, G Mellgren

**Affiliations:** 1 Institute of Medicine, University of Bergen, N-5021 Bergen, Norway; 2 The Hormone Laboratory, Haukeland University Hospital, N-5021 Bergen, Norway; 3 Section of Endocrinology, Division of Medicine, University Hospital of North Norway, N-9038 Tromsø, Norway; 4 Edinburgh Breast Unit, Western General Hospital, University of Edinburgh, Edinburgh EH4 2XU, UK; 5 Faculty Division at Akershus University Hospital, University of Oslo, N-1478 Lørenskog, Norway; 6 Department of Medicine, Section of Oncology, Akershus University Hospital, N-1478 Lørenskog, Norway; 7 Department of Oncology, Haukeland University Hospital, N-5021 Bergen, Norway

**Keywords:** steroid receptor co-activator, AIB1, PGC-1*α*, HER-2, breast cancer, aromatase inhibitors

## Abstract

**Background:**

Acquired resistance to endocrine therapy in breast cancer is poorly understood. Characterisation of the molecular response to aromatase inhibitors in breast cancer tissue may provide important information regarding development of oestrogen hypersensitivity.

**Methods:**

We examined the expression levels of nuclear receptor co-regulators, the orphan nuclear receptor liver receptor homologue-1 and HER-2/neu growth factor receptor using real-time RT-PCR before and after 13–16 weeks of primary medical treatment with the aromatase inhibitors anastrozole or letrozole.

**Results:**

mRNA expression of the steroid receptor co-activator 1 (SRC-1) and peroxisome-proliferator-activated receptor *γ* co-activator-1*α* (PGC-1*α*) was correlated (*P*=0.002), and both co-activators increased during treatment in the patient group as a whole (*P*=0.008 and *P*=0.032, respectively), as well as in the subgroup of patients achieving an objective treatment response (*P*=0.002 and *P*=0.006). Although we recorded no significant change in SRC-3/amplified in breast cancer 1 level, the expression correlated positively to the change of SRC-1 (*P*=0.002). Notably, we recorded an increase in HER-2/neu levels during therapy in the total patient group (18 out of 26; *P*=0.016), but in particular among responders (15 out of 21; *P*=0.008).

**Conclusion:**

Our results show an upregulation of co-activator mRNA and HER-2/neu during treatment with aromatase inhibitors. These mechanisms may represent an early adaption of the breast cancer cells to oestrogen deprivation *in vivo*.

Hormonal manipulations through oestrogen deprivation or administration of anti-oestrogens have a key function in breast cancer therapy. The third-generation aromatase inhibitors (AIs) have shown improved outcome compared to tamoxifen when used as adjuvant therapy and in treatment of metastatic disease in postmenopausal women (Bonneterre *et al*, 2001; Mouridsen *et al*, 2001; Howell *et al*, 2005; Jakesz *et al*, 2005; Thurlimann *et al*, 2005; Coombes *et al*, 2007). However, although first-line hormonal therapy may cause an objective response among 20–50% of patients with metastatic breast cancer and stabilise disease in many patients (Bonneterre *et al*, 2001; Mouridsen *et al*, 2001), resistance and disease progression inevitably occur.

Patients receiving neo-adjuvant treatment with the AIs experience a profound suppression of oestrogen levels, and the suppression of oestrogen in tumour is comparable with the fall in plasma oestrogen levels and *in vivo* total body aromatase inhibition (Geisler *et al*, 2001, 2002, 2008). *In vitro* models of *de novo* resistance to endocrine therapy have indicated that breast cancer cells have the ability to adapt to low oestrogen levels by developing oestrogen hypersensitivity (Lippman *et al*, 1976; Masamura *et al*, 1995; Chan *et al*, 2002) through changes in gene expression and activation of growth factor pathways (Kuang *et al*, 2005; Santen *et al*, 2005). Thus, characterisation of the molecular response to AIs in breast cancer tissue may provide important information regarding development of oestrogen hypersensitivity.

The transcriptional activity of the oestrogen receptor (ER) is regulated not only by its ligands, but also by the levels of ER co-regulators. High levels of co-activators may force ER into an active conformation that stimulates oestrogen-induced gene expression (Jordan and O’Malley, 2007). Thus, changes in the levels of ER co-activators may be of importance for the response to endocrine therapy. The expression of the steroid receptor co-activator-1 (SRC-1) has been associated with nodal positivity and endocrine resistance (Fleming *et al*, 2004; Myers *et al*, 2004). Furthermore, SRC-3/AIB1 is over-expressed in more than 30% of breast cancers with gene amplification in 5–10% of the tumours (Anzick *et al*, 1997; Murphy *et al*, 2000; List *et al*, 2001). Growth factor pathways may activate the co-activators at the posttranscriptional level (Rowan *et al*, 2000; Fleming *et al*, 2004; Wu *et al*, 2007; O’Malley *et al*, 2008), and over-expression of these co-activators, similar to over-expression of HER-2/neu, has been associated with inferior response to endocrine therapy (Osborne *et al*, 2003; Shin *et al*, 2006). It has been reported that status of some patients converts from HER-2/neu negative at the initiation of endocrine therapy to positive serum HER-2/neu at the time of progression (Lipton *et al*, 2005). However, the knowledge of HER-2/neu expression and the levels of ER co-regulators in tumour tissue during treatment with AIs is limited.

The orphan nuclear receptor liver receptor homologue-1 (LRH-1) is a specific activator of aromatase gene expression in human breast pre-adipocytes and a regulator of oestrogen biosynthesis (Clyne *et al*, 2002; Zhou *et al*, 2005). Although LRH-1 is transcriptionally regulated by ER (Annicotte *et al*, 2005) and is stimulated by co-activators, such as the peroxisome-proliferator-activated receptor *γ* co-activator-1*α* (PGC-1*α*), to execute its function (Safi *et al*, 2005), little is known about the regulation of LRH-1 and PGC-1*α* in breast tumour tissue during aromatase inhibition.

Although several studies have reported the different NR co-regulators to be over-expressed in breast cancer (Anzick *et al*, 1997; Bautista *et al*, 1998; Berns *et al*, 1998; Bouras *et al*, 2001; List *et al*, 2001; Hudelist *et al*, 2003; Iwase *et al*, 2003; Osborne *et al*, 2003; Fleming *et al*, 2004; Myers *et al*, 2004, 2005), little is known about the levels of co-regulators during oestrogen deprivation through treatment with AIs. Of interest, the level of the ER co-repressor NCoR has been shown to be downregulated in breast cancer cells resistant to anti-oestrogens (Wang *et al*, 2006). In this study, we examined the mRNA levels of the ER co-regulators SRC-1, SRC-3/AIB1, PGC-1*α*, NCoR, the nuclear receptor LRH-1 as well as the HER-2/neu growth factor receptor, together with other potential markers of endocrine response (pS2 and Ki67) and tissue oestrogen levels from the same tumours before and during treatment with the third-generation AIs anastrozole and letrozole.

## Materials and methods

### Study population

This material was collected in three similar protocols performed in Bergen, Norway (B) and Edinburgh, UK (E) as previously reported (Geisler *et al*, 2001, 2008; Miller *et al*, 2006). In summary, a total of 31 breast cancer patients treated with a non-steroidal AI (anastrozole or letrozole) as primary medical treatment (previously termed neo-adjuvant therapy) were enrolled. All patients provided written informed consent, and each protocol was approved by the local regulatory authorities.

### Treatment and tissue collection

Of 31 patients, 12 received anastrozole as an oral dose of 1 mg daily for 15 weeks (B), whereas letrozole was given as an oral dose of 2.5 mg daily with a treatment period of 16 weeks for 12 patients (B) and 13 weeks for 7 patients (E). Treatment was administered up to the day of surgery.

Tumour size was estimated by calculating the product of the largest diameter and its perpendicular. The patients were classified as responders or non-responders depending on more or less than 50% reduction in tumour size, respectively. In two of the studies (Geisler *et al*, 2001, 2008) clinical response was assessed by clinical tumour measurement using a calliper. In the third study (Miller *et al*, 2006) tumour assessment was carried out by ultrasound. Breast tumour tissue available for gene expression analysis was collected by core-cut (E) or incisional biopsy (B) before treatment and during final surgery.

### Tissue oestrogen measurements and histochemical methods

Intra-tumoural levels of E_1_, E_2_ and E_1_S were measured in the Norwegian materials using a highly sensitive HPLC-RIA method (Geisler *et al*, 2000), and are previously reported (Geisler *et al*, 2001, 2008).

ER, progesterone receptor (PR), HER-2/neu, Ki67 and the oestrogen-regulated gene *pS2* were analysed using standard immunohistochemical methods as already published (Geisler *et al*, 2001; Miller *et al*, 2006). ER and PR were reported as percentage of positively stained cells and the tumours were considered positive if ⩾10% of the cells stained for ER/PR (B), or as Allred score (E) where the first number represents an estimation of ER- or PR-positive tumour cells (0, none; 1, <1%; 2, 1–10%; 3, 10–33%; 4, 33–66% and 5, >66%) and the second number represents the average intensity of ER- or PR-positive tumours cells (0, none; 1, weak; 2, intermediate and 3, strong) (Harvey *et al*, 1999). Apoptosis was estimated using the TUNEL method (terminal deoxynucleotidyl transferase-mediated dUTP-biotin nick end labelling).

### Quantitative real-time RT-PCR

Tumour tissue was homogenised using a MagNA Lyser (50–100 mg; Roche, Basel, Switzerland) or manually (∼25 mg) and total RNA was extracted using Trizol (Invitrogen, Carlsbad, CA, USA) according to the manufacturer's recommendations. Total RNA was resuspended in PCR-grade water, and RNA quality and concentration were estimated by optical density measurement using the Nanodrop (Saveen Werner, Copenhagen, Denmark) and a Bioanalyzer (Applied Biosystems, Lincoln, CA, USA). Each sample of 1 *μ*g total RNA was reverse transcribed using the First Strand cDNA Synthesis Kit (Roche). The cDNA was stored at −20°C until use.

Real-time PCR reactions were carried out on a LightCycler 3 (Roche) using the SYBR Green detection format. Because of a marked variation in expression levels of our target genes, we calculated the expression relative to the geometric mean of two housekeeping genes: glyceraldehyde-3-phosphate dehydrogenase (*GAPDH*), which demonstrated a considerable higher mRNA-expression profile compared to the other reference gene, TATA-box binding protein (*TBP*). After each PCR run, a melting curve analysis was carried out to control for production of primer dimers and/or unwanted PCR products. An RNA standard was also included in every PCR run to control for inter-assay variation. Gene specific primers from Eurogentec (Herstal, Belgium) and TIB Molbiol (Berlin, Germany) were: SRC-1 (sense, 5′-aggcccagagccagtttac-3′; anti-sense, 5′-caggatctccgatttgatggtta-3′), SRC-3/AIB1 (sense, 5′-gaccgcttttacttcaggcatt-3′; anti-sense, 5′-tgtgttaaccaggtcctcttgct-3′), PGC-1*α* (sense, 5′-cccatttgagaacaagactat-3′; anti-sense, 5′-ggttatcttggttggcttt-3′), NCoR (sense, 5′-gatctatactcgtctcatctccgt-3′; anti-sense, 5′-agcaggctgaaggacttcc-3′), LRH-1 (sense, 5′-gctctccagcaagcatcc-3′; anti-sense, 5′-tcatttggtcatcaaccttaa-3′), HER-2/neu (sense, 5′-ccagccttcgacaacctctatt-3′; anti-sense, 5′-tgccgtaggtgtccctttg-3′), GAPDH (sense, 5′-accacagtccatgccatcac-3′; anti-sense, 5′-tccaccaccctgttgctgta-3′) and TBP (sense, 5′-tgcacaggagccaagagtgaa-3′; anti-sense, 5′-cacatcacagctccccacca-3′).

Expression levels of mRNA were estimated using external standard curves with serially diluted plasmids with known concentration for each target gene, except for HER-2/neu where serially diluted cDNA from an HER-2/neu-positive patient sample were used. Fold change in mRNA expression during treatment was calculated using the crossing point (Cp) for each sample and the efficiency (Eff) of each transcript, using the formula Eff_target gene_^ΔCp^/Eff_housekeeping gene_^ΔCp^. The fold change was estimated relative to both *GAPDH* and *TBP*, and thereafter calculated as the geometric mean of both (Pfaffl, 2001).

### Statistical analysis

The relative values of mRNA expression for all genes analysed and tissue oestrogen levels were found to be log-normally distributed. Thus, all values are presented as their geometric mean with 95% confidence intervals. Comparison between response groups was carried out using the Mann–Whitney *U*-test. Changes in gene expression during treatment were analysed using Wilcoxon signed rank test, and correlation between parameters was analysed using the Spearman rank correlation coefficient. The Ki67, pS2 and percentage of apoptotic cells are given as their arithmetic means. To reduce the number of false positives, we set the threshold *P*-value for statistical significance to 0.01. All statistical analyses were carried out using SPSS software package, version 14.0.2 (SPSS, Chicago, IL, USA).

## Results

A total of 31 patients were treated with anastrozole or letrozole as primary medical therapy for 13–16 weeks before surgery. One patient was excluded because of insufficient quantities of tissue available, leaving 30 tumour samples for gene expression analysis. Only ER-positive breast cancers were eligible for the study, but one tumour turned out to be ER negative at re-analysis with immunohistochemistry. The same tumour was the only HER-2/neu-amplified tumour in the study and was one out of six tumours that did not respond to therapy. Clinical data and tumour characteristics are summarised in Table 1.

The mRNA expression levels of the co-activators SRC-1, SRC-3/AIB1, PGC-1*α*, co-repressor NCoR, the orphan nuclear receptor LRH-1 and the HER-2/neu growth factor receptor were examined in human breast cancer tissue before and during aromatase inhibition. Noteworthy, we did not observe any significant differences in mRNA expression between anastrozole- and letrozole-treated subjects or between the subgroups treated with letrozole for 13 and 16 weeks. Analysing all patients together, the mRNA levels of our target genes were as presented in Table 2.

We observed no correlations among pre-treatment mRNA levels of the co-activators SRC-1, SRC-3/AIB1, co-repressor NCoR and the orphan nuclear receptor LRH-1. However, PGC-1*α* mRNA expression pre-treatment level correlated to change in Ki67 expression.

**Figure 1:**
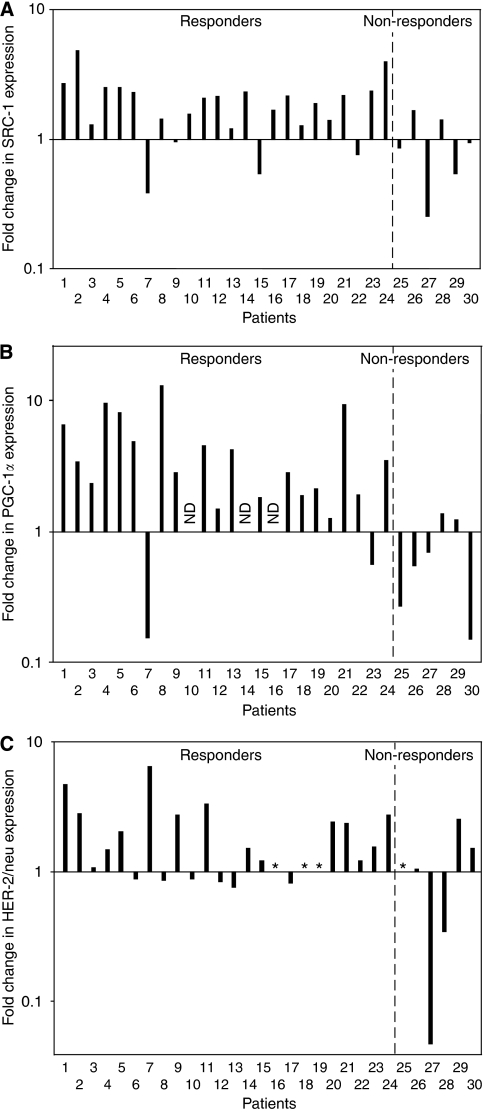
Individual fold changes in mRNA expression of SRC-1 (**A**), PGC-1*α* (**B**) and HER-2/neu (**C**) in patients during oestrogen deprivation. RNA was purified from the same breast tumour in the individual breast cancer patient before and after 13–16 weeks of treatment with either letrozole or anastrozole. Fold change in mRNA expression was estimated using real-time RT-PCR and presented relative to the housekeeping genes *GAPDH* and *TBP*. Calculations are based on the crossing point (Cp) for each sample and the efficiency (Eff) of each transcript, using the formula Eff_target gene_^ΔCp^/Eff_housekeeping gene_^ΔCp^. Patients marked as not detected (n.d.) had Cp outside the detection limit. Patients marked by ^*^ are excluded due to insufficient tumour tissue left for analysis.

During treatment, the mRNA levels of SRC-1 were upregulated in 22 of 30 subjects (Figure 1A) with a mean fold change of 1.40 (*P*=0.008). A non-significant increase in SRC-3/AIB1 mRNA expression was also observed (mean change of 1.32; *P*=0.090). PGC-1*α* mRNA increased in 21 out of 27 tumours expressing detectable levels at baseline, but the observed change in the total patient group was not statistical significant (Figure 1B; mean change of 1.91; *P*=0.032). No significant change in expression of NCoR or LRH-1 was detected. HER-2/neu mRNA was upregulated in 18 out of 26 tumours (Figure 1C) with a mean fold change of 1.35 (borderline significance; *P*=0.016). The overall fold changes in SRC-1 mRNA expression correlated positively to expression of PGC-1*α* (*R*=0.565, *P*=0.002) as well as SRC-3/AIB1 (*R*=0.551, *P*=0.002).

**Figure 2:**
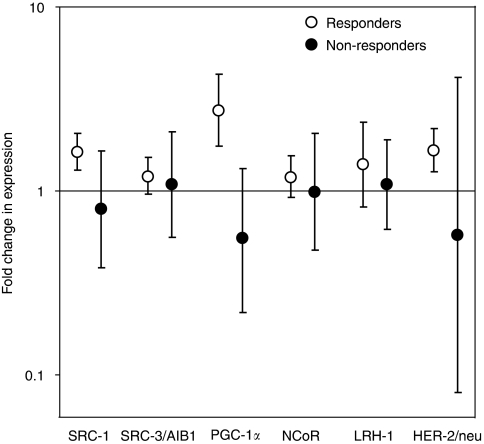
Fold change in mRNA expression of target genes during oestrogen deprivation. RNA was purified from tumours from breast cancer patients before and after 13–16 weeks of treatment with either letrozole or anastrozole. Patients were classified as responders (R) or non-responders (NR) based on clinical tumour measurements during treatment. SRC-1 (R, *n*=24; NR, *n*=6), SRC-3/AIB1 (R, *n*=23; NR, *n*=6), PGC-1*α* (R, *n*=21; NR, *n*=6), NCoR (R, *n*=24; NR, *n*=6), LRH-1 (R, *n*=19; NR, *n*=6) and HER-2/neu (R, *n*=21; NR, *n*=5) mRNA expression was estimated using real-time RT-PCR. Fold change in mRNA expression during treatment was calculated using the crossing point (Cp) for each sample and the efficiency (Eff) of each transcript, using the formula Eff_target gene_^ΔCp^/Eff_housekeeping gene_^ΔCp^. The fold changes are presented as geometric mean with 95% confidence interval of the fold change calculated relative to the housekeeping genes *GAPDH* and *TBP*. Differences in fold changes between R and NR were analysed by the Mann–Whitney *U*-test: SRC-1; *P*=0.023, SRC-3/AIB1; *P*=0.667, PGC-1*α*; *P*=0.002, NCoR; *P*=0.437, LRH-1; *P*=0.227 and HER-2/neu; *P*=0.186.

Among responders, SRC-1 was upregulated in 20 out of 24 tumours (Figure 1A, mean change of 1.62; *P*=0.002). This change during treatment appeared to be different compared to the mean change of 0.79 among six non-responders (*P*=0.023). Pre-treatment levels of PGC-1*α* mRNA were higher in tumours not responding compared to those responding to therapy (borderline significance; *P*=0.012). Notably, PGC-1*α* mRNA was upregulated in 19 out of 21 responders (mean change of 2.75; *P*=0.006). This increase was significantly different from the mean change of 0.54 in non-responders (*P*=0.002). HER-2/neu mRNA was upregulated in 15 out of 21 responders that were available for analysis (Figure 1C) with a significant mean fold change of 1.66 (*P*=0.008). This increase in responders appeared to be different compared to the mean change of 0.58 among non-responders (*P*=0.186). However, as for changes in mRNA expression of SRC-1, SRC-3/AIB1, NCoR and LRH-1, the difference between responders and non-responders was not significant (Figure 2).

Intra-tumoural concentrations of E_1_, E_2_ and E_1_S and biomarkers, including Ki67, pS2 and apoptotic cells, are presented in Table 3. As expected, PR, pS2 and Ki67 decreased significantly during treatment in both responders and non-responders, with a more profound decrease of Ki67 among responders. Except for a non-significant positive correlation between SRC-1 and LRH-1 mRNA expression and the expression of Ki67 during treatment (*R*=−0.502, *R*=−0.648 and *P*=0.040, *P*=0.017, respectively), no correlation between changes in mRNA levels and expression of pS2, Ki67 or degree of oestrogen suppression was recorded (data not shown).

## Discussion

Changes in gene expression profiles in breast cancer tumours during endocrine treatment have been reported before (Kristensen *et al*, 2005; Mackay *et al*, 2007; Miller *et al*, 2007, 2009; Harvell *et al*, 2008). To our knowledge this is the first study evaluating expression of nuclear receptor co-regulators in breast cancer patients during oestrogen deprivation. The ER co-activators SRC-1 and SRC-3/AIB1 have been previously linked to endocrine sensitivity in breast cancer. High levels of SRC-1 and/or SRC-3/AIB1 are suggested to be associated with insensitivity to treatment with tamoxifen (Myers *et al*, 2004), and HER-2/neu over-expression confers an inferior response to treatment with tamoxifen as well as to AIs (Shin *et al*, 2006; Dowsett *et al*, 2008; Rasmussen *et al*, 2008).

We observed a significant increase in SRC-1 and PGC-1*α* mRNA expression in response to treatment with two third-generation non-steroidal AIs. SRC-1 and PGC-1*α* enhance ER activity, and increased levels may sensitise cells to oestrogens at lower concentrations (Lonard *et al*, 2007).

Several groups have reported MCF-7 cells exposed to E_2_ at low concentrations over time to develop a state of hypersensitivity, achieving growth stimulation at a hormone concentration 1 out of 1000 to 1 out of 10 000 the concentration required for wild-type cells (Lippman *et al*, 1976; Masamura *et al*, 1995; Martin *et al*, 2003). Although several mechanisms, including activation of the insulin-like growth factor receptor and mitogen-activated protein kinases, have been provided (Santen *et al*, 2005; Sabnis *et al*, 2007), some data indicate HER-2/neu may be important (Song *et al*, 2002; Martin *et al*, 2003, 2005). Still we lack scientific evidence showing that such mechanisms actually occur *in vivo* and can be related to therapy resistance. Although our data should be interpreted with care, to our knowledge they represent the first evidence of mechanisms that could possibly sensitise tumour cells to oestrogen stimulation in response to aromatase inhibition *in vivo*. The upregulation of co-activators and HER-2/neu was evident in treatment responsive tumours in contrast to no significant changes in the subgroup of non-responsive tumours. Our observations may suggest activation of regulatory mechanisms in response to E_2_ suppression in endocrine-sensitive cells that are absent or less active in tumours insensitive to hormonal manipulation. Thus, one possible hypothesis is that the increasing levels of SRC-1 and PGC-1*α* represent a cellular response to AIs and that the increase in co-activator levels may reflect the efficiency of endocrine therapy. At the same time, changes in gene expression that could potentially lead to increased oestrogen sensitivity could be one of several mechanisms contributing to acquired therapy resistance evolving over time. However, because the non-responders represent a small subgroup in this study, the data concerning the subgroups should be interpreted with caution.

Even though the levels of PGC-1*α* for all patients were more than a 100-fold lower compared to the other co-activators, pre-treatment PGC-1*α* expression did most clearly separate between the responding groups with a 4-fold higher geometric mean value among non-responders compared to responders. PGC-1*α* is known to interact with SRC-1 for full transcriptional activity (Puigserver *et al*, 1999; Bourdoncle *et al*, 2005), and it is an important regulator of LRH-1 and peroxisome-proliferator-activated receptor *γ* (Puigserver *et al*, 1999; Safi *et al*, 2005).

LRH-1 is suggested to exert oncogenic effects through effects on aromatase expression and cell-cycle regulators such as G_1_-phase cyclins (Clyne *et al*, 2002; Annicotte *et al*, 2005; Zhou *et al*, 2005). We observed no changes of LRH-1 mRNA during treatment, which would suggest that local aromatase upregulation by this transcriptional activator may not be a response to oestrogen deprivation. On the other hand, it is well known that the transcriptional activity of LRH-1 is stimulated through interaction with the SRCs (Xu *et al*, 2004). Thus, even though the LRH-1 mRNA expression is unaffected during oestrogen deprivation, this does not rule out that the transcriptional activity of LRH-1 is increased.

A significant increase in HER-2/neu expression was observed during therapy among tumours responsive to AIs. The serum level of HER-2/neu has been shown to be increased from baseline to time of progression in approximately 25% of patients treated with letrozole or tamoxifen (Lipton *et al*, 2005). In addition, *in vitro* studies have shown that oestrogen deprivation is associated with an increase in HER-2/neu expression (Dati *et al*, 1990; Read *et al*, 1990; Warri *et al*, 1991). Recently, it was reported that letrozole upregulates the protein level of HER-2/neu in MCF-7Ca xenografts in mice despite continued response to treatment, and it has been hypothesised that an inverse relationship exists between HER-2/neu and ER*α* (Sabnis *et al*, 2009). In line with this, it has been shown that HER-2/neu transcription can be repressed by oestrogen (Bates and Hurst, 1997). A recent report shows that the transcription factor-paired box 2 gene product (PAX2) and SRC-3/AIB1 compete for binding and regulation of HER-2/neu transcription in MCF-7 cells (Hurtado *et al*, 2008). High levels of SRC-3/AIB1 outcompete PAX2 leading to an increase in the HER-2/neu transcription after tamoxifen treatment (Hurtado *et al*, 2008). SRC-3/AIB1 has also been shown to be required for HER-2/neu oncogenic activity (Fereshteh *et al*, 2008). Even though we did not observe a correlation between changes in SRC-3/AIB1 and HER-2/neu mRNA expression in our study, the expression of both genes increased during oestrogen deprivation. It is possible that the enhanced level of HER-2/neu mRNA could be explained by loss of repression due to an increase in SRC-3/AIB1 or other ER co-activators. Interestingly, it has recently been reported that patients with ER-positive and HER-2/neu-negative breast cancer with a poor response to tamoxifen may obtain an increased time to progression by having the tyrosine kinase inhibitor lapatinib added to letrozole (Johnston *et al*, 2009). Thus, an increase in HER-2/neu expression may represent a circumvention of oestrogen deprivation.

In this study we have focused on accurate quantifications of mRNA expression, but it should be noted that changes in mRNA and protein expression are not always similar, and one should keep in mind that post-translational modifications and regulation of protein turnover rates may also affect the co-regulator protein levels (Greenbaum *et al*, 2003).

In conclusion, the results from this study with unique matched pre- and on-treatment samples suggest that co-activators and HER-2/neu are upregulated in tumours during AI therapy as an early response to effective oestrogen deprivation. Increasing levels of co-activator mRNA may represent a response by the cells to antagonise the oestrogen deprivation effect of anastrozole and letrozole.

## Figures and Tables

**Table 1:** Treatment and tumour characteristics for the patients

**Patient**	**Treatment**	**Hospital**	**Treatment period**	**ER^a^**	**PR^a^**	**Response^b^**	**HER-2/neu**
1	Letrozole	B^c^	16	100	30/40	R	Neg.
2	Letrozole	B	16	>50	>50	R	Neg.
3	Letrozole	B	16	70/80	10	R	Neg.
4	Letrozole	B	16	80	<10	R	NA
5	Letrozole	B	16	100	100	R	Neg.
6	Letrozole	B	16	100	100	R	Neg.
7	Letrozole	B	16	100	100	R	Neg.
8	Letrozole	B	16	>50	>50	R	Neg.
9	Letrozole	B	16	100	100	R	Neg.
10	Letrozole	B	16	100	50	R	Neg.
11	Letrozole	B	16	80/100	0/20	R	Neg.
12	Letrozole	B	16	>80	>80	R	Neg.
13	Letrozole	E	13	5+3	1+2	R	NA
14	Letrozole	E	13	5+3	4+2	R	NA
15	Letrozole	E	13	5+3	3+3	R	NA
16	Letrozole	E	13	5+2	5+2	R	NA
17	Anastrozole	B	15	86	53	R	Neg.
18	Anastrozole	B	15	93	0	R	Neg.
19	Anastrozole	B	15	83	84	R	Neg.
20	Letrozole	E	13	5+3	5+3	R	NA
21	Letrozole	E	13	5+3	5+3	R	NA
22	Anastrozole	B	15	98	0	R	Neg.
23	Anastrozole	B	15	92	86	R	Neg.
24	Anastrozole	B	15	87	70	R	Neg.
25	Letrozole	E	13	5+3	5+3	NR	NA
26	Anastrozole	B	15	92	79	NR	Neg.
27	Anastrozole	B	15	NA	NA	NR	Neg.
28	Anastrozole	B	15	91	86	NR	Neg.
29	Anastrozole	B	15	2	0	NR	Pos.
30	Anastrozole	B	15	82	7.5	NR	Neg.

Abbreviations: NA=not available; Neg.=negative; Pos.=positive; R=responders; NR=non-responders.

a Expressed as percentage of cells staining positively (IHC) or as an Allred score (Harvey *et al*, 1999).

b Classification of treatment response was based on change in tumour size calculated as a product of the largest diameter and its perpendicular and subjects presented as R or NR.

c Patients were treated at Haukeland University Hospital, Bergen (B) or Edinburgh Breast Unit Western General Hospital (E).

**Table 2:** Influence of anastrozole and letrozole on SRC-1, SRC-3/AIB1, PGC-1*α*, NCoR, LRH-1 and HER-2/neu mRNA expression

	**Pre-treatment**	**On treatment**	**Fold change**	***P* for change^a^**	***P* between subgroups^b^**
*SRC-1*					
All patients	0.2961 (0.2059–0.4260)^c^	0.4034 (0.2583–0.6301)^d^	1.40 (1.11–1.79)	0.008	0.023
Responders	0.3576 (0.2496–0.5123)	0.5585 (0.3888–0.8023)	1.62 (1.29–2.04)	0.002	
Non-responders	0.1391 (0.0419–0.4622)	0.1098 (0.0220–0.5484)	0.79 (0.38–1.65)	0.463	
					
*SRC-3/AIB1*					
All patients	0.3682 (0.3053–0.4440)	0.4367 (0.3562–0.5354)	1.32 (1.08–1.62)	0.090	0.667
Responders	0.3482 (0.2832–0.4281)	0.4223 (0.3344–0.5334)	1.21 (0.97–1.52)	0.078	
Non-responders	0.4562 (0.2606–0.7985)	0.4969 (0.2803–0.8810)	1.09 (0.56–2.10)	0.753	
					
*PGC-1α*					
All patients	0.0023 (0.0014–0.0037)^e^	0.0040 (0.0027–0.0058)	1.91 (1.20–3.05)	0.032	0.002
Responders	0.0017 (0.0011–0.0027)	0.0041 (0.0028–0.0061)	2.75 (1.74–4.34)	0.006	
Non-responders	0.0072 (0.0021–0.0245)	0.0037 (0.0009–0.0150)	0.54 (0.22–1.31)	0.116	
					
*NCoR*					
All patients	0.2028 (0.1447–0.2842)	0.2342 (0.1756–0.2842)	1.16 (0.92–1.46)	0.245	0.437
Responders	0.1897 (0.1264–0.2848)	0.2280 (0.1609–0.3231)	1.20 (0.93–1.56)	0.162	
Non-responders	0.2651 (0.1398–0.5026)	0.2607 (0.1465–0.4639)	0.98 (0.47–2.05)	0.753	
					
*LRH-1*					
All patients	0.0127 (0.0082–0.0196)	0.0167 (0.0129–0.0216)	1.31 (0.87–1.98)	0.074	0.227
Responders	0.0117 (0.0067–0.0205)	0.0162 (0.0120–0.0219)	1.39 (0.81–2.38)	0.091	
Non-responders	0.0169 (0.0093–0.0307)	0.0184 (0.0088–0.0383)	1.09 (0.62–1.90)	0.753	
					
*HER-2/neu*					
All patients	0.5388 (0.3780–0.7681)	0.8288 (0.5213–1.3180)	1.35 (0.94–1.94)	0.016	0.186
Responders	0.4777 (0.3525–0.6525)	0.8659 (0.5721–1.3106)	1.66 (1.27–2.18)	0.008	
Non-responders	1.6320 (0.4482–5.9430)	0.9298 (0.0702–12.3108)	0.58 (0.08–4.13)	0.893	

a Pre-treatment levels were compared to on-treatment levels after 13–16 weeks using the Wilcoxon 2 group test.

b Comparisons of mRNA-levels between responders and non-responders during treatment were performed using the Mann–Whitney *U*-test.

c All the relative values are given as geometric means with 95% confidence intervals.

d Exclusively for SRC-1, on-treatment levels are significant different between response groups *P*=0.009 (Mann–Whitey *U*-test).

e Exclusively for PGC-1*α*, pre-treatment levels are in borderline significant different between response groups, *P*=0.012 (Mann–Whitey *U*-test).

**Table 3:** Change in biomarker and oestrogen levels during treatment with AIs

	**Pre-treatment**	**On treatment^a^ (%)**
E_1_^b^	208.6	12.1
E_2_^b^	311.5	4.9
E_1_S^b^	112.3	15.4
Ki67^c^	17.0	34.1
pS2^c^	45.3	28.0
Apoptotic cells^c^	1.7	64.7

a On treatment levels are presented as percentage of pre-treatment levels.

b Intra-tumoural levels of E_1_, E_2_ and E_1_S (fmol/g) measured by an HPLC-RIA method and given as geometric means.

c Ki67 and pS2 measured by immunohistochemistry and percentage of apoptotic cells analysed by the TUNEL method and results given as their arithmetic means.
